# All-arthroscopic treatment of tibial avulsion fractures of the posterior cruciate ligament

**DOI:** 10.3205/iprs000081

**Published:** 2016-01-18

**Authors:** Clemens Gwinner, Arnd Hoburg, Sophie Wilde, Imke Schatka, Björn Dirk Krapohl, Tobias M. Jung

**Affiliations:** 1 Center for Musculoskeletal Surgery, Charité – Medical University of Berlin, Germany; 2 Institute for Radiology and Nuclear Medicine, Charité – Medical University of Berlin, Germany; 3 Department of Plastic and Hand Surgery, St. Marien-Krankenhaus Berlin, Germany; 4 Center for Musculoskeletal Surgery, Charité – University Medicine Berlin, Germany

**Keywords:** posterior cruciate ligament, tibial avulsion fracture, Tight Rope

## Abstract

**Background:** The posterior cruciate ligament (PCL) avulsion fracture from its tibial insertion is a rare condition. Despite the further technical advent in refixation of avulsion fractures, the reported failure rate of current approaches remains high and the optimal surgical technique has not been elucidated yet.

The purpose of the current study is to present an all-inside arthroscopic reconstruction technique for bony tibial avulsion fractures of the PCL and initial clinical outcomes.

**Methods:** Patients underwent a thorough clinical and radiological examination of both knees at 3, 6, 12, 18, and if possible also at 24 months.

Clinical evaluation included subjective and objective IKDC 2000, Lysholm score, and KOOS score. Radiographic imaging studies included CT scans for assessment of osseous integration and anatomic reduction of the bony avulsion. In addition to that posterior stress radiographs of both knees using the Telos device (Arthrex, Naples, USA) were conducted to measure posterior tibial translation.

**Results:** A total of four patients (1 female, 3 male; ø 38 (± 18) years), who underwent arthroscopic refixation of a PCL avulsion fracture using the Tight Rope device were enrolled in this study. Mean follow up was 22 [18–24] months.

The mean subjective IKDC was 72.6% (± 9.9%). Regarding the objective IKDC three patients accounted for grade A, one patient for grade C. The Lysholm score yielded 82 (± 6.9) points. The KOOS score reached 75% (± 13%; symptoms 76%, pain 81%, function 76%, sports 66%, QoL 64%).

All patients showed complete osseous integration and anatomic reduction of the bony avulsion. The mean posterior tibial translation at final follow up was 2.8 [0–7] mm.

**Conclusions:** All-arthroscopic treatment of tibial avulsion fractures of the posterior cruciate ligament provides satisfactory clinical results in a preliminary patient cohort. It is a reproducible technique, which minimizes soft tissue damage and obviates a second surgery for hardware removal. Further clinical studies with larger patient cohorts and a control group are needed to further confirm these preliminary results.

## Zusammenfassung

**Hintergrund:** Knöcherne Ausrisse des hinteren Kreuzbandes (HKB) stellen eine seltene Verletzungsentität dar. Eine zeitnahe Versorgung wird als notwendig erachtet. Bisher gibt es jedoch keinen allgemeingültigen Therapiealgorithmus oder eine standardisierte Operationsmethode. Ziel der vorliegenden Studie ist es, erste Ergebnisse einer rein-arthroskopischen Operationstechnik vorzustellen, welche intraartikuläre Begleitverletzungen adressiert und eine Zweitoperation zur Implantatentfernung vermeidet.

**Material und Methoden:** Alle Patienten wurden nach 3, 6, 12, 18 und ggf. nach 24 Monaten klinisch und radiologisch nachuntersucht. Klinisch wurden IKDC 2000, KOOS und der Lysholm Score erhoben. Radiologisch wurden gehaltene Röntgenstressaufnahmen (Telos-Halteappererat) durchgeführt, um die posteriore tibiale Translation zu quantifizieren. Außerdem wurde nach 6 Monaten eine Computertomografie des betroffenen Kniegelenks durchgeführt, um die knöcherne Konsolidierung und anatomische Reposition zu überprüfen.

**Ergebnisse:** Bisher konnten vier konsekutive Patienten (3m, 1w; ø 38 (±18) Jahre) mit einem Nachuntersuchungszeitraum von ø 22 [18–24] Monaten in die Studie eingeschlossen werden. Der durchschnittliche subjektive IKDC betrug 72,6% (± 9,9%). Drei Patienten erreichten Grad A und eine Patientin Grad C im objektiven IKDC. Der Lysholm Score betrug 82 (± 6,9) Punkte und der KOOS Score ergab 75% (± 13%; Symptome 76%, Schmerz 81%, Funktion 76%, Sport 66%, QoL 64%). Bei allen Patienten zeigte sich in der CT-Diagnostik eine annähernd anatomische Konsolidierung. Die gehaltenen Stressaufnahmen zeigten zum Zeitpunkt des Follow-up eine vermehrte posteriore, tibiale Translation im Vergleich zur gesunden Gegenseite von ø 2,8 [0–7] mm.

**Schlussfolgerung:** Die rein-arthroskopische Versorgung von knöchernen Ausrissen des hinteren Kreuzbandes in transtibialer Tight-Rope-Technik (Arthrex, Naples, USA) zeigt positive klinische und radiologische Frühergebnisse in einem bisher kleinen Patientenkollektiv. Auf diese Weise kann der komplikationsbehaftete dorsale Zugang zum Kniegelenk sowie eine Zweitoperation zur Implantatentfernung vermieden werden.

## Introduction

Avulsion fractures of the tibial insertion of the posterior cruciate ligament (PCL) are an infrequent injury in the Western world. Notably, its incidence is much higher in countries like India or China with more frequent two-wheeler related injuries [1]. The PCL serves as a primary restraint against posterior tibial translation and adjusts rotational movement in near extension. Once injured, a malfunctioning posterior cruciate ligament carries the potential for long-term sequelae, including persistent instability, decreased activity level and degenerative changes [2], [3], [4].

According to current literature, an early surgical treatment of displaced bony avulsion of the PCL is regarded necessary in order to restore knee stability and to prevent mal- or non-union [4], [5], [6]. Several surgical techniques of open reduction and internal screw or Kirschner wire fixation with direct visualization via a posterior approach have been regarded as suitable [1]. 

Due to its deep location and the complexity of the adjacent anatomy, minimally invasive and arthroscopic techniques are gaining interest [7], [8]. This growing popularity has prompted a rapid increase in arthroscopic techniques, albeit a paucity of studies exists regarding their clinical outcome. Despite comparable biomechanical properties of open and arthroscopic techniques, direct clinical advantages and disadvantages have yet not been fully elucidated [9].

The aim of the current study is to determine preliminary clinical and radiological parameters of an all-arthroscopic approach for isolated PCL avulsion fractures using the Tight Rope device (Arthrex, Naples, USA) [10].

## Material and methods

Inclusion criteria comprised patients with tibial avulsion fractures type II or III as described by White et al. [11]. Only fractures within the first three weeks after injuries were included. Contrarily, we excluded patients, whose radiographs did not include a true lateral view of the tibia as determined by proper superimposition of the femoral condyles. All patients had to give informed consent to participate. The minimum follow up period was two years.

Diagnosis in all cases was established by history, clinical examination and radiographic evaluation. Knee radiographs in standing anteroposterior, standing lateral and a computertomography were performed to further delineate the fracture pattern. Posterior stress radiography (GA II stress device; Telos, Weiterstadt, Germany) was not used in the acute setting to prevent additional dislocation of the bony avulsion.

### Surgical technique

The patient is placed in supine position and after a thorough physical examination a tourniquet is applied to the patient’s thigh. Preoperative intravenous antibiotics are administered and the leg is prepped and draped in a sterile fashion. An initial diagnostic arthroscopy is performed, using the routine high anterolateral and anteromedial portals. Additional posteromedial portals are created as needed to obtain visual access to the tibial fracture site of the posterior cruciate ligament. The posterior capsule is partly detached from its tibial footprint, frayed soft tissue is debrided and the extent of the bony avulsion is documented. Subsequent to the diagnostic arthroscopy, a 15 mm long pretibial incision is performed about 10 mm distal to the tibial tuberosity. A tibial PCL guide (Fa. Storz, Tuttlingen, Germany) is introduced and the avulsion fracture is reduced by direct arthroscopic visualization. A 2.4 mm guidewire is inserted aiming for the midpart of the avulsion. Additional guidewires to provide temporary reduction are applied according to the size of the bony avulsion. After that, the central guidewire is overdrilled with a 4.5 mm cannulated drill. Guidewire placement and drilling direction are both controlled fluoroscopically. Once the dorsal cortex is perforated, a Tight Rope is shuttled transtibially and pulled through the bony avulsion. Terminatory, the Tight Rope is flipped under arthroscopic guidance and traction is applied to the pretibial sutures. With the tibia drawn anteriorly, the Tight Rope is tightened until complete reduction is achieved. 

### Rehabilitation

Postoperative care consists of immobilization of the operated knee in a straight PTS splint (posterior tibial support; Medi, Bayreuth, Germany) in order to avoid posterior tibial translation for a total of four weeks. The patient is encouraged to start isometric quadriceps contraction immediately after surgery. Partial weight bearing and gradual passive mobilization is performed during this period in prone position. Adjoining, free passive range of motion and full weight bearing is gradually established and active range of motion exercises initiated. At this stage, the straight PTS splint is changed into a controlled hinge PCL orthesis, which again generates an anterior directed force on the proximal aspect of the tibia to avoid posterior tibial translation for another six weeks. Return to sports and heavy labor is performed at six months according to the patient’s functional recovery.

### Clinical and radiological assessment

Clinical and radiological evaluation was obtained preoperatively, after three, and six months as well as one and two years after surgery, subsequently. At the time of follow up, the same advanced musculoskeletal physician, who was not part of the initial surgical team, performed a thorough physical examination of both knees. Clinical evaluation was assessed by the subjective and objective IKDC 2000, Lysholm score and the KOOS score. 

**Figure 1:**
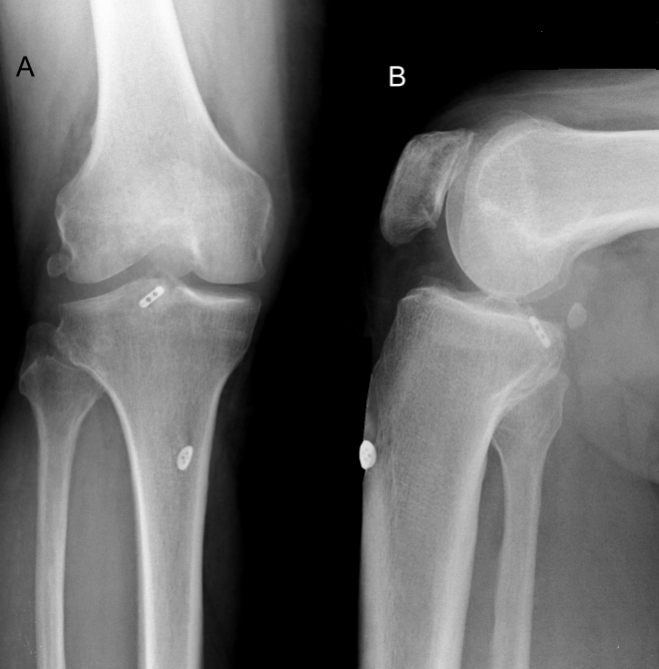
Anteroposterior (A) and lateral (B) radiographs six month after surgery showing anatomically reduction and union of the bony avulsion

**Figure 2:**
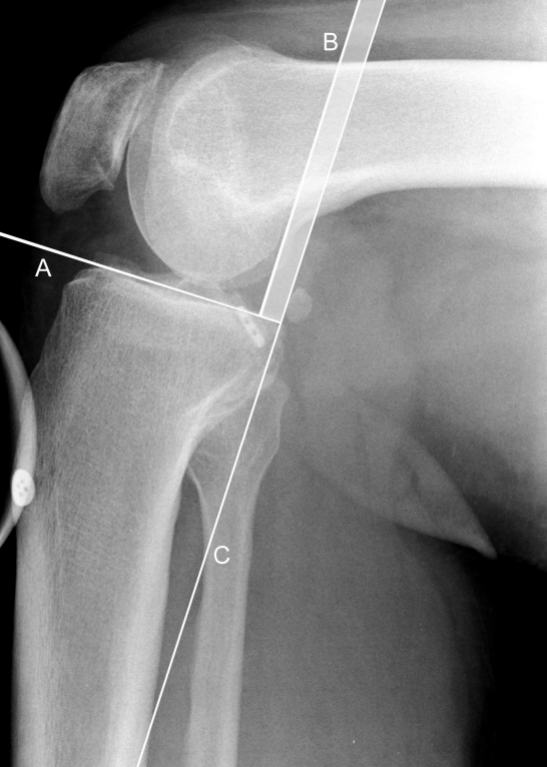
Measurement of the posterior tibial translation, using Telos stress device; as described by [12], [13]. Perpendicular to the tangent of the tibial plateau (A), the midpoints between the most posterior contours of the medial and lateral femoral condyles (B) and tibial plateaus (C) are established. The distance between these two points is regarded as the posterior tibial translation and is subsequently assessed in relation to that of the uninjured contralateral knee.

Radiographic imaging studies included CT scans and anteroposterior as well as posterior stress radiographs of both knees (Figure 1 (Fig. 1)). Anatomic healing of the bony avulsions and posterior tibial translation were evaluated by an advanced musculoskeletal radiologist using a PACS workstation (Centricity RIS-I 4.2 Plus, GE Healthcare, Milwaukee, WI, USA). Posterior tibial translation was measured by the use of the Telos device at 90° of flexion with a posteriorly directed force of 150 N at the level of the tibial tubercle. Posterior tibial displacement was measured according to Jacobsen [12] as well as Staubli and Jakob [13] (Figure 2 (Fig. 2)).

## Results

Four patients (1 female, 3 male; 41.5 (± 13.8) years) met our inclusion criteria and were enrolled in this study. All patients were evaluated by clinical and radiological examination at a mean time of follow up of 22 [18–24] months. 

**Figure 3:**
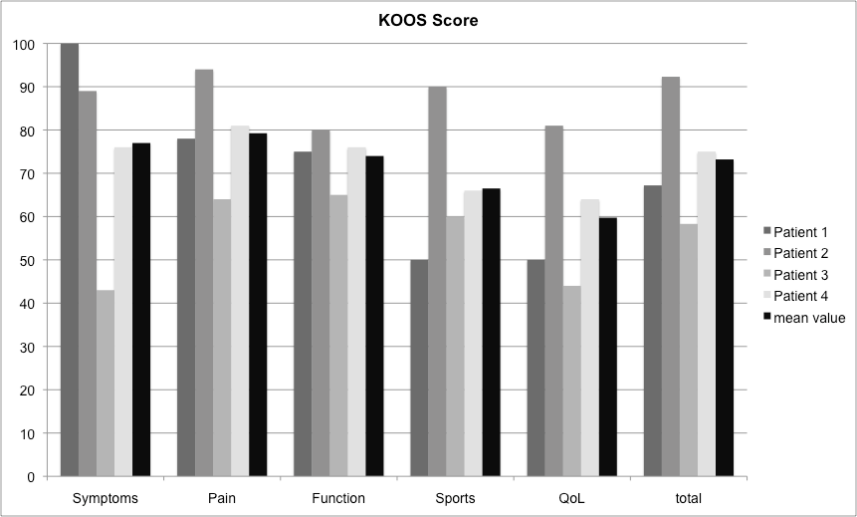
KOOS score [%] of the patient cohort

At time of follow-up, mean subjective IKDC was 72.6% (± 9.9%). Regarding the objective IKDC, three patients accounted for grade A, one patient for grade C. The Lysholm score yielded 82 (± 6.9) points. The KOOS score reached 75% (± 13%; symptoms 76%, pain 81%, function 76%, sports 66%, QoL 64%), respectively (Figure 3 (Fig. 3)). There were no complications, such as neurovascular injuries, superficial or deep infections. Two patients underwent additional meniscal surgery within the initial arthroscopy.

Radiological assessment confirmed proper reduction and complete osseous healing of the bony avulsion in all cases. The mean posterior tibial translation was 2.8 (0–7) mm at time of follow up. Three out of the four patients returned to their pre-injury level of knee function and sports. Notably, no revision surgery or implant removal had to be performed. 

## Discussion

The results of the study indicate that the presented all-arthroscopic technique using a Tight Rope device appears to be a feasible and reproducible alternative for PCL avulsion fractures.

Even though bony avulsions only account for a small sub-group of all PCL injuries, accurate reduction seems mandatory to enable physiological joint biomechanics and to prevent further osteoarthritic progression and to minimize failure of concomitant meniscal or ligament repair. 

According to current literature, two approaches are mainly used for the surgical treatment of displaced tibial avulsion fractures of the PCL; an open and an arthroscopic one. Both appear to have advantages and disadvantages. Previous literature commonly recommended an open approach using the traditional posterior, posterolateral or the posteromedial approach [14]. However, open exposure of the bony avulsion can be difficult as a result of the anatomical insertion site of the PCL deep within the posterior tibial plateau and the close proximity of the popliteal neurovascular bundle. Furthermore, postoperative scar tissue and inevitably weakness of the gastrocnemius muscle may restrict postoperative range of motion [14]. 

Recently, arthroscopic approaches are gaining interest, as they are able to address concomitant lesions, such as chondral or meniscal tears. In addition, they are less invasive due to decreased exposure of the posterior capsule or muscle and therefore reducing both soft tissue damage and postoperative scar formation. However, arthroscopic approaches are far from being accepted. 

In 1988 Martinez-Moreno and Blanco-Blanco were the first to describe a percutaneous fixation technique under arthroscopic guidance [15]. Even though their technique has never been reported as a clinical application, they were soon followed by Littlejohn and Geissler, who presented an arthroscopically, percutaneous fixation technique in a clinical setting [16]. Hereinafter arthroscopic techniques using suture anchors or suture fixation were established [5], [7], [17], [18], [19], [20].

More recently, transtibial shuttle techniques were introduced with encouraging results [21], [22]. Both authors concluded that the assessed all-inside suspensory devices present an easy to handle technique, which offers a homogenous distribution of pressure to the avulsion site and can be extended to fragments of any size. On the basis of the growing popularity of the Tight Rope device in AC joint repair [23], [24] the presented technique has been implemented [10].

The findings of the current study complement the work of the aforementioned authors and the presented techniques offers several advantages. Firstly, it obviates hardware removal. Additionally, it appears to be a reproducible technique and reduction of the bony avulsion can be assessed both by fluoroscopy and arthroscopic guidance. Due to the arthroscopic procedure, it allows for inspection and treatment of concomitant injuries, as required in our patient cohort. Furthermore, the fracture can be measured suitably and interposed tissue can be removed from the fracture gap. Even in cases of a comminuted fracture pattern the Tight Rope device appears to be suitable, due to the broad tibial insertion site of the PCL. 

The present study has several limitations. The small number of patients has to be taken into consideration as it limits the ability to draw firm conclusions. Against this background the presented results should be regarded as preliminary and should mainly proof the feasibility and reproducibility.

However, there are also some limitations to the presented technique. One of the disadvantages is that the 4.5 mm drill hole might break smaller or thinner bony avulsions. Thus the size of the avulsion should be measured carefully prior surgery. Especially in patients with significant osteoporosis, further research is required to determine whether or not this method is suitable. Another drawback is, that due to the bony reduction, this technique is not able to address occult intraligamentous injuries of the posterior cruciate ligament. Inoue et al. [25] stated that occult midsubstance injuries do not significantly affect postoperative posterior translation of the affected knee. Nevertheless, one patient had a side-to-side difference of 7 mm in posterior tibial translation, even though she had documented anatomical healing of the tibial insertion of the posterior cruciate ligament, which leaves room for further discussion of occult midsubstance injuries in bony ligament avulsions.

Future studies could overcome these shortfalls if aiming at a larger patient cohort and if using a control group, which undergoes fracture reduction via an open approach. Due to the PCL attachment deep within the popliteal fossa, arthroscopic fixation can be both challenging and demanding [26]. Additionally, the adherence of the PCL to the posterior capsule increases the risk to embed the joint capsule or local fat tissue into the fracture gap. 

Due to the inferior visualization of an open approach, arthroscopic surgery of bony avulsions of the PCL provides superior anatomic reduction and union of the fracture fragments with decreased morbidity. Of note, it is mandatory from our perspective, that the surgeon is experienced in creating posterior arthroscopic portals and approaching the posterior compartment. 

In conclusion, the presented all-arthroscopic treatment of PCL avulsion fractures is feasible and results in good clinical outcomes with well-documented radiographic healing. It is a reproducible technique, which minimizes soft tissue damage and obviates a second surgery for hardware removal. Further clinical studies, with larger patient cohorts and a long-term follow up are needed to further confirm these preliminary results.

## Notes

### Competing interests

The authors declare that they have no competing interests.

## Acknowledgements

We thank the Institute for Radiology of the Charité – Medical University of Berlin for their continuous support and image allocation.

## References

[1] Bali K, Prabhakar S, Saini U, Dhillon MS (2012). Open reduction and internal fixation of isolated PCL fossa avulsion fractures. Knee Surg Sports Traumatol Arthrosc.

[2] Gill TJ, DeFrate LE, Wang C, Carey CT, Zayontz S, Zarins B, Li G (2004). The effect of posterior cruciate ligament reconstruction on patellofemoral contact pressures in the knee joint under simulated muscle loads. Am J Sports Med.

[3] Boynton MD, Tietjens BR (1996). Long-term followup of the untreated isolated posterior cruciate ligament-deficient knee. Am J Sports Med.

[4] Logan M, Williams A, Lavelle J, Gedroyc W, Freeman M (2004). The effect of posterior cruciate ligament deficiency on knee kinematics. Am J Sports Med.

[5] Huang W, Gong X, Rahul M, Priyanka S, Wang C, Liang X, Ding G, Hu N (2015). Anterior arthroscopic-assisted fixation of posterior cruciate ligament avulsion fractures. Eur J Med Res.

[6] Jung TM, Höher J, Weiler A (2006). Screw fixation of a 4 1/2-year-old PCL avulsion injury. Knee Surg Sports Traumatol Arthrosc.

[7] Chen SY, Cheng CY, Chang SS, Tsai MC, Chiu CH, Chen AC, Chan YS (2012). Arthroscopic suture fixation for avulsion fractures in the tibial attachment of the posterior cruciate ligament. Arthroscopy.

[8] Chen W, Tang D, Kang L, Ding Z, Sha M, Hong J (2012). Effects of microendoscopy-assisted reduction and screw fixation through a single mini-incision on posterior cruciate ligament tibial avulsion fracture. Arch Orthop Trauma Surg.

[9] Sasaki SU, da Mota e Albuquerque RF, Amatuzzi MM, Pereira CA (2007). Open screw fixation versus arthroscopic suture fixation of tibial posterior cruciate ligament avulsion injuries: a mechanical comparison. Arthroscopy.

[10] Gwinner C, Kopf S, Hoburg A, Haas NP, Jung TM (2014). Arthroscopic Treatment of Acute Tibial Avulsion Fracture of the Posterior Cruciate Ligament Using the TightRope Fixation Device. Arthrosc Tech.

[11] White EA, Patel DB, Matcuk GR, Forrester DM, Lundquist RB, Hatch GF 3rd, Vangsness CT, Gottsegen CJ (2013). Cruciate ligament avulsion fractures: anatomy, biomechanics, injury patterns, and approach to management. Emerg Radiol.

[12] Jacobsen K (1976). Stress radiographical measurement of the anteroposterior, medial and lateral stability of the knee joint. Acta Orthop Scand.

[13] Stäubli HU, Jakob RP (1990). Posterior instability of the knee near extension. A clinical and stress radiographic analysis of acute injuries of the posterior cruciate ligament. J Bone Joint Surg Br.

[14] Jazayeri SM, Esmaili Jah AA, Karami M (2009). A safe postero-medial approach to posterior cruciate ligament avulsion fracture. Knee Surg Sports Traumatol Arthrosc.

[15] Martinez-Moreno JL, Blanco-Blanco E (1988). Avulsion fractures of the posterior cruciate ligament of the knee. An experimental percutaneous rigid fixation technique under arthroscopic control. Clin Orthop Relat Res.

[16] Littlejohn SG, Geissler WB (1995). Arthroscopic repair of a posterior cruciate ligament avulsion. Arthroscopy.

[17] Kim SJ, Shin SJ, Choi NH, Cho SK (2001). Arthroscopically assisted treatment of avulsion fractures of the posterior cruciate ligament from the tibia. J Bone Joint Surg Am.

[18] Kim SJ, Shin SJ, Cho SK, Kim HK (2001). Arthroscopic suture fixation for bony avulsion of the posterior cruciate ligament. Arthroscopy.

[19] Choi NH, Kim SJ (1997). Arthroscopic reduction and fixation of bony avulsion of the posterior cruciate ligament of the tibia. Arthroscopy.

[20] Gui J, Wang L, Jiang Y, Wang Q, Yu Z, Gu Q (2009). Single-tunnel suture fixation of posterior cruciate ligament avulsion fracture. Arthroscopy.

[21] Horas U, Meissner SA, Heiss C, Schnettler R (2010). Arthroscopic fixation of posterior cruciate ligament avulsion fractures: a new minimally invasive technique. Knee Surg Sports Traumatol Arthrosc.

[22] Wajsfisz A, Makridis KG, Van Den Steene JY, Djian P (2012). Fixation of posterior cruciate ligament avulsion fracture with the use of a suspensory fixation. Knee Surg Sports Traumatol Arthrosc.

[23] Scheibel M, Ifesanya A, Pauly S, Haas NP (2008). Arthroscopically assisted coracoclavicular ligament reconstruction for chronic acromioclavicular joint instability. Arch Orthop Trauma Surg.

[24] Gerhardt C, Kraus N, Pauly S, Scheibel M (2013). Arthroskopisch assistierte Stabilisierung akuter Schultereckgelenkverletzungen in Doppel-TightRope™-Technik : Einjahresergebnisse. Unfallchirurg.

[25] Inoue M, Yasuda K, Kondo E, Saito K, Ishibe M (2004). Primary repair of posterior cruciate ligament avulsion fracture: the effect of occult injury in the midsubstance on postoperative instability. Am J Sports Med.

[26] Zhang X, Cai G, Xu J, Wang K (2013). A minimally invasive postero-medial approach with suture anchors for isolated tibial avulsion fracture of the posterior cruciate ligament. Knee.

